# Clinical Outcomes in Maximum Tolerated Medical Therapy in Penetrating Keratoplasty for Bullous Keratopathy

**DOI:** 10.3389/fmed.2022.810848

**Published:** 2022-03-01

**Authors:** Seoyoung Wy, Young Kook Kim, Jin Wook Jeoung, Mee Kum Kim

**Affiliations:** ^1^Department of Ophthalmology, Seoul National University College of Medicine, Seoul, South Korea; ^2^Department of Ophthalmology, Seoul National University Hospital, Seoul, South Korea; ^3^Laboratory of Ocular Regenerative Medicine and Immunology, Biomedical Research Institute, Seoul National University Hospital, Seoul, South Korea

**Keywords:** Ahmed glaucoma valve, cornea, endothelial cell, maximum tolerated medical therapy, penetrating keratoplasty

## Abstract

**Purpose:**

To compare the clinical outcomes of maximum tolerated medical therapy (MTMT) in patients with penetrating keratoplasty (PKP) with those of Ahmed glaucoma valve (AGV) implantation.

**Methods:**

The medical records were retrospectively reviewed in patients who had undergone PKP for bullous keratopathy and were treated with MTMT or AGV implantation for the management of glaucoma. A total of 18 bullous keratopathic patients were investigated between January 2010 and February 2017: 9 patients treated with MTMT and 9 patients treated with AGV implantation. Non-corrected visual acuity (NCVA), intraocular pressure (IOP), endothelial cell density (ECD), hexagonality, coefficient of variation (CV), central corneal thickness (CCT), median survival time of the graft, and the presence of epithelial keratopathy were compared between the groups at each time point or between baseline and after treatment of glaucoma in each group.

**Results:**

There were no significant differences in the visual acuity and corneal thickness between the two groups or within each group over time. Both groups showed a significant reduction in IOP compared with the baseline IOP, and IOP reductions were greater in the AGV group than in the MTMT group (*p* = 0.040). Significant ECD reductions were found in each group between the baseline and 6 months (*p* = 0.008 in the MTMT group, *p* = 0.015 in the AGV group); however, no differences were found between the two groups until 12 months. The significant hexagonality reduction was found in the AGV group between the baseline and 12 months (*p* = 0.018). The median survival time showed no significant difference in the survival analysis.

**Conclusions:**

Maximum tolerated medical therapy in penetrating keratoplasty for bullous keratopathy seems to similarly affect the endothelial cell density or graft survival when compared with at least 12 month-followed Ahmed glaucoma valve implantation.

## Introduction

Glaucoma is a well-known risk factor for corneal endothelial decompensation after penetrating keratoplasty (1–4). Both uncontrolled intraocular pressure (IOP) and Ahmed glaucoma valve (AGV) implantation may affect the endothelial cell decompensation of the graft (1, 5). To avoid endothelial damage associated with AGV implantation, maximum tolerated medical therapy (MTMT) can be maintained in patients with glaucoma after penetrating keratoplasty (PKP). Although the graft survival with anti-glaucoma medication was reported to be lower in PKP than that without glaucoma (6), the long-term effect of MTMT on the graft survival or endothelial cell density (ECD) has not been clearly established.

Evidence suggests that the topical anti-glaucoma medication is not directly involved in the reduction of ECD in patients with glaucoma patients (7) or corneal donor grafts (8). However, the long-term use of topical anti-glaucoma medication induces significant ocular surface changes in patients who underwent PKP, such as dry eye, superficial punctate keratitis, conjunctival scarring, and ocular surface inflammation (9–13), which may affect not only the success of subsequent glaucoma surgery but also graft survival. These toxic effects on the ocular surface may be attributed to multiple factors, such as preservatives, low pH, or active ingredients *per se* (11). Ocular surface damage with topical anti-glaucoma medication may lead to poor compliance, resulting in either less-controlled or fluctuated IOP. Given recent confocal microscopic findings showing a significant increase in the density of dendritic cells with topical anti-glaucoma medication (9), the long-term MTMT may also affect the graft rejection in the early period. Considering that the rapid reduction of ECD in PKP (7.5%/year) compared with that in normal cornea (0.6%/year) over time (14, 15), MTMT may also affect the overall graft survival in the compromised cornea due to PKP. Therefore, this study aimed to compare the effect of MTMT with that of AGV implantation on the changes in endothelial cell density (ECD), hexagonality, CV, or the survival of grafts in patients who underwent PKP for a bullous keratopathy.

## Materials and Methods

### Patient Selection

This retrospective study was approved by the Institutional Review Board of Seoul National University College of Medicine (IRB No. 1905-038-1031, Seoul, South Korea) and adhered to the tenets of the Declaration of Helsinki. We retrospectively reviewed medical records of patients who were treated with PKP, such as re-PKP, for bullous keratopathy and MTMT or AGV implantation for the management of glaucoma following PKP. The study included patients who were followed-up for at least 12 months after MTMT or AGV implantation at the cornea clinic in Seoul National University Hospital between January 2010 and February 2017. The choice of AGV implantation was determined by the clinical judgment of two glaucoma specialists (Y. K. Kim and J. W. Jeoung), when the IOP was unacceptably high even with MTMT based on the visual field defect status and the severity/or progression of the optic nerve damage in each patient. The exclusion criteria were as follows: the patients who had diagnosed with other corneal disease with glaucoma, who had undergone any other intraocular surgeries within 6 months before PKP, who had undergone any other intraocular surgeries between PKP and AGV implantation, who had undergone any other intraocular surgeries during MTMT in PKP, and who had not regular examination.

The study included a total of 18 patients: 9 patients who had MTMT and 9 patients who underwent AGV implantation. The demographics of the patients in each group were shown in Table 1. The patients who had undergone re-PKP before AGV were also included in both groups (3 patients in the MTMT group and 1 patient in the AGV group). The proportions of re-PKPs did not show a statistically significant difference between the groups. The mean age of patients in the MTMT and AGV groups was 66.1 years (47–79 years) and 60.9 years (35–75 years), respectively. The mean time intervals between PKP and the onset of anti-glaucoma treatment were 12.4 months (3–23 months) and 23.8 months (1–58 months) in the MTMT and the AGV groups (*p* > 0.05), respectively. The mean follow-up durations were 36.6 months (4–67 months) and 36.9 months (4–113 months) in the MTMT and the AGV groups, respectively. There were no statistically significant differences in all demographic characteristics.

**Table 1:** Demographics of the patients in the MTMT and AGV groups.

	**MTMT**	**AGV**	*P-*value^*^
Number of patients	9	9	
Age (years)	66.1 ± 12.4 (47–79)	60.9 ± 12.8 (35–73)	0.489
Sex (male: female)	8: 1	7: 2	0.527
Laterality (right: left)	4: 5	5: 4	0.637
No. of re-PKP	3 (33.3%)	1 (11%)	0.576
Time interval between PKP and onset of anti-glaucoma treatment (months)	12.4 ± 9.9 (3–23)	23.8 ± 21.7 (1–58)	0.340
Follow-up duration (months)	34.2 ± 17.8 (4–67)	50.4 ± 40.0 (4–113)	0.546

* *Using the Mann–Whitney test or the Fisher's exact test or the Chi-square test where appropriate*.

*Data was presented as Mean ± SD (ranges)*.

*MTMT, maximum tolerated medical therapy; AGV, Ahmed glaucoma valve; PKP, penetrating keratoplasty*.

### Surgical Procedures and MTMT

Penetrating keratoplasty was conducted by a single corneal surgeon (M. K. Kim) with 10-0 interrupted sutures, and the lens extraction combined with intraocular lens insertion was performed in 1 eye in the AGV group.

Ahmed glaucoma valve implantation was performed on the superotemporal aspect of each eye. A traction suture through the clear cornea was used in the upper peripheral cornea to enhance the exposure to the surgical field. A 5-mm circumferential and vertical incision was made in the conjunctiva and Tenon's capsule, 1 mm posterior to the corneal limbus, followed by a dissection between the Tenon's capsule and the sclera. The body of the AGV (model S2 with a surface area of 184 mm^2^; New World Medical, Rancho Cucamonga, CA, USA) was inserted under the Tenon's capsule between the superior rectus muscle and the lateral rectus muscle. The body of the AGV was fixed to the sclera by two 8-0 prolene (Ethicon, Inc.) anchoring sutures at the front edge of the plate bilaterally, 8–9 mm from the corneal limbus. An anterior chamber puncture, parallel with the iris surface, was made 1 mm posterior to the corneal limbus, using a 23-gauge needle. A silicone tube was cut and ~2 mm was inserted into the anterior chamber, in a bevel-up position. The silicon tube near the corneal limbus was covered using a 4 mm × 3 mm half-thickness sclera flap. The surgery was completed using continuous running sutures of the Tenon's capsule and the conjunctiva.

Maximum tolerated medical therapy was defined as the use of two or more of the following medications for lowering IOP; topical carbonic anhydrase inhibitor (CAI) (2% dorzolamide or 1% brinzolamide), topical beta-blocker (0.5% timolol), topical alpha-agonist (0.15% brimonidine, or 0.2% brimonidine, or 0.5% apraclonidine), topical prostaglandin analog (0.005% latanoprost or 0.004% travoprost), and oral administration of acetazolamide. Table 2 summarizes the representative types of combinations of anti-glaucoma medications used in each group. The number of anti-glaucoma medications was not statistically different between those two groups.

**Table 2:** The types of combinations of anti-glaucoma medications used in the MTMT and AGV groups.

**Anti-glaucoma medications**	**MTMT**	**AGV**	*P*-value^*^
The types of combinations	2% dorzolamide/0.5% timolol + 0.15% brimonidine + 0.005% latanoprost + oral acetazolamide	2% dorzolamide/0.5% timolol + 0.15% brimonidine + 0.005% latanoprost + oral acetazolamide	
	2% dorzolamide/0.5% timolol + 0.15% brimonidine + 0.004% travoprost + oral acetazolamide	1% brinzolamide/0.5% timolol + 0.15% brimonidine + 0.005% latanoprost + oral acetazolamide	
	2% dorzolamide/0.5% timolol + 0.5% apraclonidine + 0.004% travoprost + oral acetazolamide	2% dorzolamide + 0.2% brimonidine/0.5% timolol + 0.004% travoprost + oral acetazolamide	
Mean number	3.5 ± 1.3 (2–5)	4.1 ± 0.9 (3–5)	0.387

* *Using the Mann–Whitney test*.

*MTMT, maximum tolerated medical therapy; AGV, Ahmed glaucoma valve*.

### Clinical Evaluation

Data, such as demographics, type of glaucoma treatment, non-corrected visual acuity (NCVA), IOP, ECD, central corneal thickness (CCT), number of anti-glaucoma medications, presence of epithelial keratopathy, and graft survival, were collected. ECD was measured *via* a non-contact specular microscopy (SP-8800, Konan, Hyogo, Japan). CCT was measured using a pachymeter (Pocket II, Quantel Medical, Paris, France), and IOP was measured using a rebound tonometer (Icare® PRO, Icare Finland Oy, Helsinki, Finland). Graft failure was assessed through slit-lamp examination by the cornea specialist who performed PKP. We defined “graft failure” as the persistent corneal edema when the corneal edema did not disappear within 2 months of immunosuppressive treatment.

### Data Analysis

Each parameter was evaluated before anti-glaucoma treatment (AGV implantation vs. MTMT) and at intervals of 6 and 12 months after the anti-glaucoma treatment. NCVA, IOP, ECD, CCT, and number of anti-glaucoma medications were compared between the AGV and MTMT groups at each time point (inter-group analysis), and compared between the baseline and 6 or 12 months after the anti-glaucoma treatment in each group (intra-group analysis). Graft survival was analyzed and compared between the two groups.

### Statistical Analysis

A statistical analysis was performed using SPSS 20.0 (SPSS Inc., Chicago, IL, USA). The Mann–Whitney test was used to compare each of the parameters at the baseline and after treatment between AGV and MTMT groups and categorical variables were compared using Fisher's exact test or chi-square test (inter-group analysis). The Wilcoxon signed-rank test was used to compare parameters between the baseline and after treatment in each group (intra-group analysis). Graft survival was analyzed using the Kaplan–Meier method to estimate the median survival time (MST). The log-rank test was used to assess the significant differences in MST between the groups. A value of *p* < 0.05 was considered statistically significant for all tests.

## Results

Table 3 summarizes baseline parameters in each group before AGV implantation. The mean baseline IOPs were 24.0 (range, 15–42) and 32.6 (range, 21–56) mmHg in the MTMT and the AGV groups (*p* > 0.05), respectively. The mean size of the donor graft was 7.88 ± 0.18 (range, 7.25–8.25) mm in the MTMT group, and 7.72 ± 0.29 (range, 7.75–8.25) mm in the AGV group, which showed no statistical significance. There were no statistically significant differences in ECD, CV, and hexagonality between those two groups.

**Table 3:** Clinical characteristics in the MTMT and AGV groups before AGV implantation.

	**MTMT**	**AGV**	*P-*value^*^
Baseline ocular parameters			
Visual acuity (logMAR)	1.6 ± 1.1 (0.7–3.7)	1.6 ± 1.0 (0.7–3.7)	0.931
Intraocular pressure (mmHg)	24.0 ± 8.6 (15–42)	32.6 ± 10.3 (21–56)	0.063
Endothelial cell density (/mm^2^)	1683.4 ± 531.3 (1,259–2,564)	1879.8 ± 1096.9 (343–3,663)	0.605
Central corneal thickness (μm)	538.1 ± 42.8 (465–595)	556.7 ± 59.6 (466–908)	0.931
Coefficient of variation	36.8 ± 5.4 (25–43)	34.0 ± 8.2 (23–46)	0.409
Hexagonality (%)	57.7 ± 10.2 (42–74)	55.0 ± 13.5 (33–67)	0.644
Lens status (phakia: pseudophakia: aphakia)	0: 7: 2	1: 8: 0	0.471
Factors associated with PKP			
Combined cataract extraction	0 (0%)	1 (11%)^+^	0.303
Donor graft size (mm)	7.88 ± 0.18 (7.75–8.25)	7.72 ± 0.29 (7.25–8.25)	0.190

* *Using the Mann–Whitney test or the Fisher's exact test or the chi-square test where appropriate*.

+ *PKP combined with simultaneous cataract surgery was undergone 3 years prior to AGV implantation*.

*Data were presented as mean ± SD (ranges)*.

*MTMT, maximum tolerated medical therapy; AGV, Ahmed glaucoma valve; PKP, penetrating keratoplasty*.

**Figure 1:**
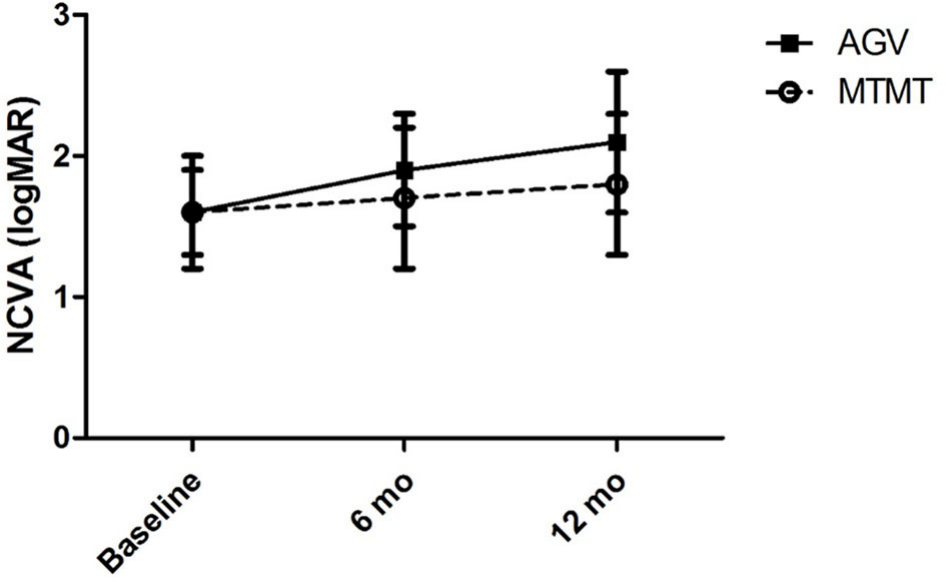
Changes in the non-corrected visual acuity (NCVA) after glaucoma treatment. No significant difference was found between the two groups, and between before and after the treatment in each group.

The visual acuities before the anti-glaucoma treatment, and at 6 and 12 months after the treatment are presented in Figure 1. The mean visual acuities before the treatment, and at 6 and 12 months after the treatment were 1.6 ± 1.1 (range, 0.7–3.7), 1.7 ± 1.4 (range, 0.4–4.7), and 1.8 ± 1.5 (range, 0.4–4.7) logMAR units in the MTMT group, and 1.6 ± 1.0 (range, 0.7–3.7), 1.9 ± 1.3 (range, 0.5–4.7), and 2.1 ± 1.4 (range, 0.5–4.7) logMAR units in the AGV group. Although, the average VA was worsened by 0.2 logMAR units after MTMT and by 0.5 logMAR units after AGV, there was no statistically significant difference in each group over the treatment or between two groups at each time point.

**Figure 2:**
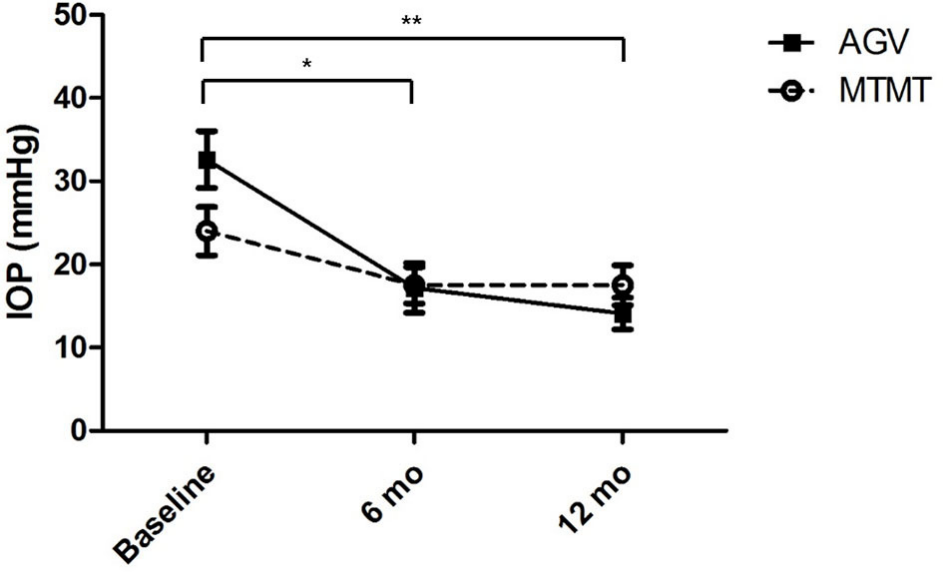
Changes in intraocular pressure (IOP) after anti-glaucoma treatment. No significant difference was found between the two groups. There were significant IOP reductions in the AGV group between baseline and 6 or 12 months after the treatment (*p = 0.020, **p = 0.008; Wilcoxon signed-rank test for intra-group analysis).

The changes in IOP are presented in Figure 2. The mean IOP before, and at 6 and 12 months after the treatment were 24.0 ± 8.6 (range, 15–42), 17.5 ± 6.6 (range, 10–32), and 17.5 ± 7.3 (range, 10–33) mmHg in the MTMT group and 32.6 ± 10.3 (range, 21–56), 17.2 ± 5.8 (range, 10–39), and 14.1 ± 5.6 (range, 7–26) mmHg in the AGV group, respectively. After AGV implantation, there were significant IOP reductions between the baseline and post-operative 6 months (*p* = 0.020) and between the baseline and post-operative 12 months (*p* = 0.008). IOP reductions were greater in the AGV group than in the MTMT group (*p* = 0.040).

**Figure 3:**
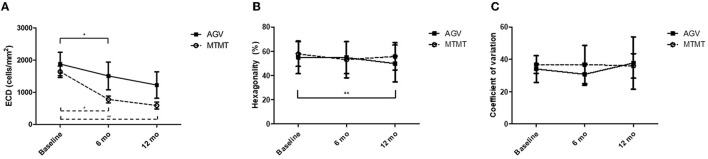
Changes in corneal endothelial cell measurements after anti-glaucoma treatment. **(A)** Changes in the endothelial cell density (ECD). No significant difference was found between the two groups. There was a significant ECD reduction in the AGV group between baseline and 6 months. There were significant ECD reductions in the AGV group between baseline and 6 and 12 months after the treatment (*p = 0.015, ^#^*p* = 0.008, ^##^*p* = 0.008; Wilcoxon signed-rank test for intra-group analysis). **(B)** Changes in hexagonality. No significant difference was found between the two groups. There was a significant hexagonality reduction in the AGV group between baseline and 12 months after the treatment in the AGV group (**p = 0.018; Wilcoxon signed-rank test). **(C)** Changes in coefficient of variation. No significant difference was found between the two groups, and between before and after the treatment in each group.

**Figure 4:**
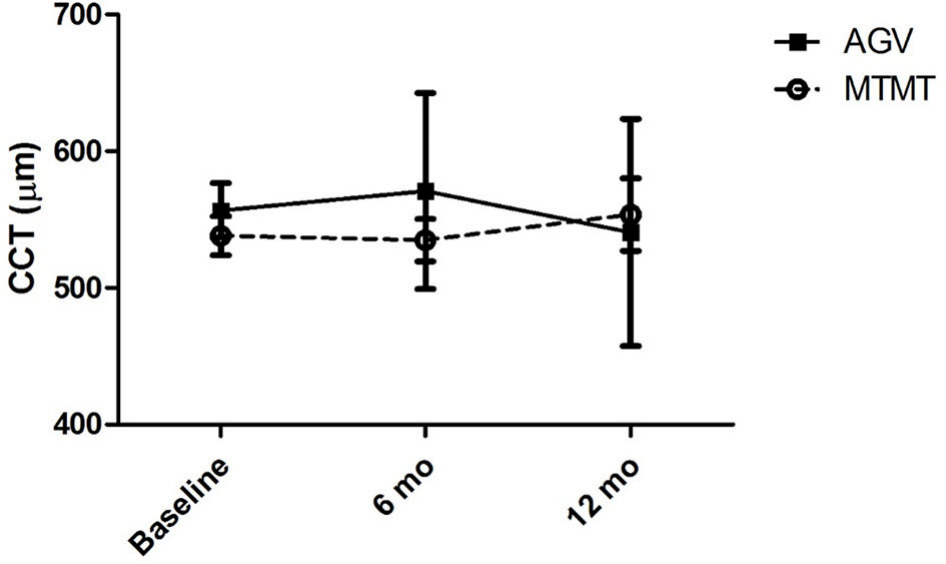
Changes in central corneal thickness (CCT) after anti-glaucoma treatment. No significant difference was found between the two groups, and between before and after the treatment in each group.

The changes in ECD, hexagonality, CV, and central corneal thickness between the two groups are presented in Figures 3, 4. The mean ECD before, and at 6 and 12 months after the treatment were 1,638.4 ± 531.3 (range, 1,259–2,564), 777.5 ± 321.7 (range 372–1,404), and 588.8 ± 324.5 (range 331–1,262) cells/mm^2^ in the MTMT group, and 1,879.8 ± 1,096.9 (range, 343–3,663), 1,509.3 ± 1,291.6 (range, 504–3,703), and 1,226.0 ± 1,227.7 (range, 606–3,333) cells/mm^2^ in the AGV group, respectively. There were significant ECD reductions between the baseline and 6 months after the treatment in both groups (MTMT, *p* = 0.008; AGV, *p* = 0.015), and between the baseline and 12 months after the treatment in the MTMT group (*p* = 0.008). Notably, there were no statistically significant differences between two groups over time. The mean hexagonality before, and at 6 and 12 months after the treatment were 57.7 ± 10.2 (range, 42–74), 53.0 ± 15.0 (range, 30–70), and 55.8 ± 11.3 (40–71)% in the MTMT group, and 55.0 ± 13.5 (range, 33–67), 54.8 ± 13.2 (range, 33–66), and 49.9 ± 15.2 (range, 35–75)% in the AGV group. There was a significant hexagonality reduction between the baseline and 12 months after the treatment in the AGV group (*p* = 0.018), but there were no statistically significant differences in hexagonality between the two groups. The mean CV before, and at 6 and 12 months after the treatment were 36.8 ± 5.4 (range, 25–43), 36.8 ± 11.9 (range 26–64), and 35.9 ± 7.6 (range, 40–71) in the MTMT group, and 34.0 ± 8.2 (range, 23–46), 30.8 ± 6.7 (range, 22–46), and 37.7 ± 16.2 (range, 26–72) in the AGV group. There were no statistically significant differences in CV before treatment, and at 6 and 12 months after the treatment in each group and between two groups. The mean corneal thickness before, and at 6 and 12 months after the treatment were 538.1 ± 42.8 (range, 465–595, 534.8 ± 46.4 (range, 470–623), and 553.6 ± 79.5 (range, 460–642) μm in the MTMT group, and 556.7 ± 59.6 (range, 493–697), 570.8 ± 108.2 (range, 449–807), and 540.6 ± 83.9 (range, 412–908) μm in the AGV group, respectively. There were no statistically significant differences in corneal thickness before the treatment, and at 6 and 12 months after the treatment in each group and between two groups.

**Figure 5:**
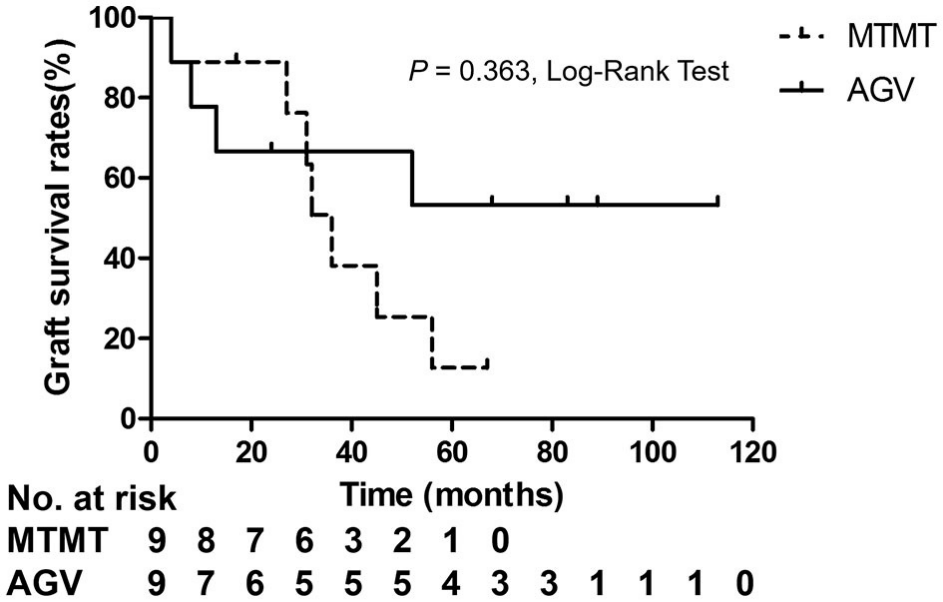
Kaplan–Meier analysis showed no significant differences in graft survival between the two groups.

Corneal graft failure occurred in 7 patients (77.7%) in the MTMT group and 3 patients (33.3%) in the AGV group eventually. There were no statistically significant differences between the two groups. The median survival time (MST) was 1,050.0 ± 575.1 (range, 120–2,010) days in the MTMT group, and 1,513.3 ± 1201.4 (range, 120–3,390) days in the AGV group, which showed no significant difference in the survival analysis (*p* = 0.363, log-rank test) (Figure 5). Superficial punctate epithelial keratopathy of the corneal grafts occurred in 10 patients (77.7%) in the MTMT group and 3 patients (33.3%) in the AGV group, and there was no statistically significant difference between the two groups (Table 4).

**Table 4:** Superficial punctate epithelial keratopathy of the corneal grafts in the MTMT and AGV groups.

		**MTMT**	**AGV**	*P-*value^*^
Superficial punctate epithelial keratopathy (No. of patients)	Yes	7	3	
	No	2	6	
Percentages (%)		77.7	33.3	0.153

* *Using the Fisher's exact test*.

*MTMT, maximum tolerated medical therapy; AGV, Ahmed glaucoma valve*.

The mean number of anti-glaucoma medications before the treatment and at 12 months after the treatment were 3.5 (range, 2–5) and 3.0 (range, 2–5) in the MTMT group and 4.1 (range, 3–5) and 1.3 (range, 0–5) in the AGV group (Table 5). There were no differences in the mean number of medications between two groups before the treatment (*p* = 0.387), while the number of medications was significantly lower in the AGV group than in the MTMT group at 12 months (*p* = 0.024).

**Table 5:** Number of topical medications in the MTMT and AGV groups.

	**MTMT**	**AGV**	*P-*value^*^
Pre-treatment	3.5 ± 1.3 (2–5)	4.1 ± 0.9 (3–5)	0.387
At 12 months	3.0 ± 1.2 (2–5)	1.3 ± 1.7 (0–5)	0.024

* *Using the Mann–Whitney test*.

*MTMT, maximum tolerated medical therapy; AGV, Ahmed glaucoma valve*.

## Discussion

In this study, effects of AGV implantation were comparable to effects of MTMT on ECD changes and graft survival in patients with penetrating keratoplasty for at least 12 months. However, surprisingly, the reduction in ECD with MTMT of PKP eyes for bullous keratopathy was greater than those eyes with normal ECD in previous studies (5, 7, 16). This is the first report to evaluate ECD changes with MTMT in corneal transplanted eyes so far. In previous reports, they described the effect of each anti-glaucoma drug on ECD, neither the combined anti-glaucoma drugs nor MTMT. Therefore, it is still noteworthy to report that the effect on ECD can be influenced by both multiple combined topical anti-glaucoma drugs and compromised endothelial cells in the host owing to the transplantation and previous history of bullous keratopathy. In the AGV group, a patient who had undergone PKP combined with simultaneous cataract surgery 3 years prior to AGV implantation was included. Since previous studies have reported that the decrease in ECD after 6 months after cataract surgery was not significant (17–19), the fact that PKP with simultaneous cataract surgery was included in the AGV group would not have affected the outcome of ECD.

Based on previous reports that suggested a graft failure of 8~26% with AGV implantation at 12 months in patients with penetrating keratoplasty (2, 20–22), our data showed a graft failure of 33% with AGV implantation, which were comparable with previous reports. The mean loss of ECD was 653/mm^2^/year in the AGV, which was comparable with previous reports (−315 cells/mm^2^/year) (21) and a recent study has suggested ECD loss of −519.97 cells/mm^2^ for 2 years (23). Given that the patients who showed unacceptably high IOP based on their severity or progression of the optic nerve damages or visual field defects had undergone AGV implantation, high pre-operative IOP in AGV, although it was insignificant, may also affect changes of ECD. In this study, hexagonality of corneal endothelial cells significantly decreased after 1 year of AGV implantation, whereas a previous study showed no significant difference in hexagonality after AGV implantation or compared with the control group (24).

**Figure 6:**
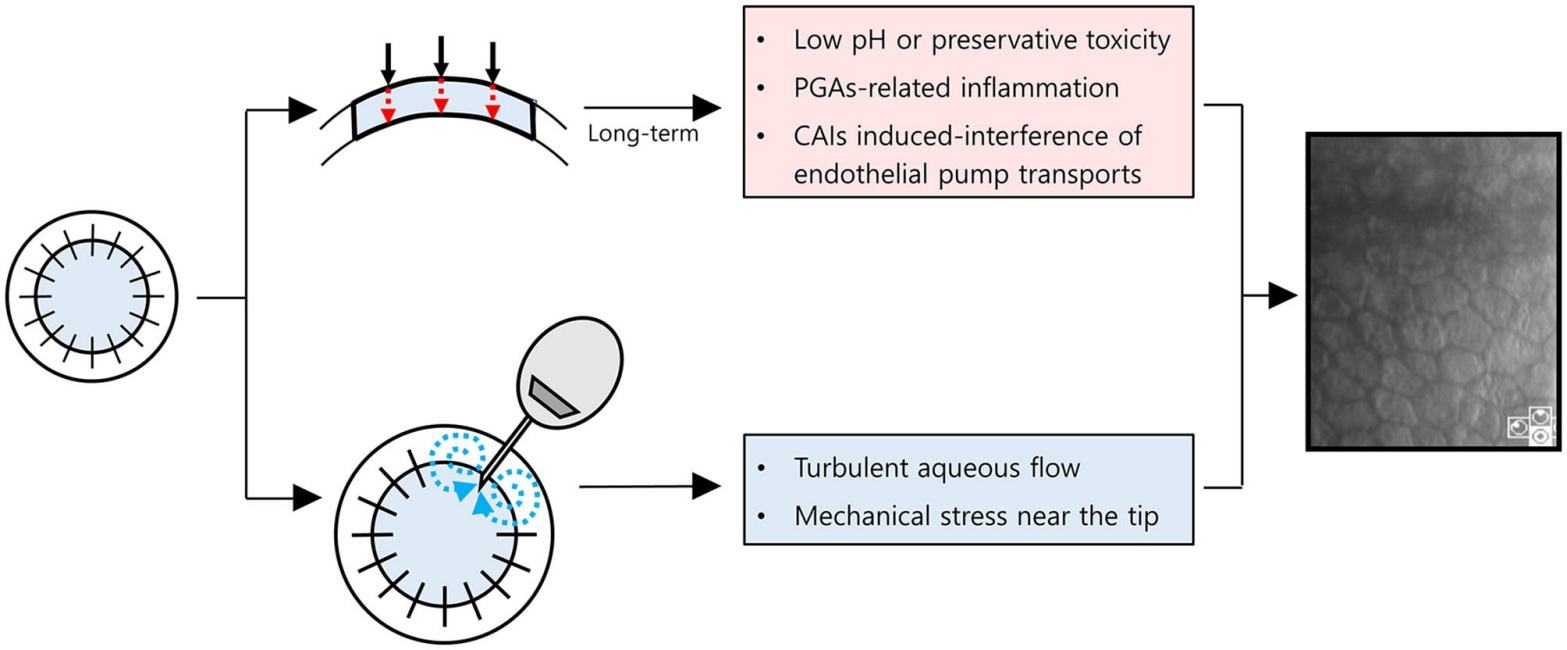
Schematic diagram for a hypothesis on how to damage corneal endothelial cells with maximum tolerated medical therapy or glaucoma drainage device. Red dot arrows indicate anti-glaucoma eyedrops are permeable to endothelial cells with (1) low pH or preservative toxicity, (2) PGAs-related inflammation, or (3) CAIs induced-interference of endothelial pump transports. Blue dot arrows indicate turbulent aqueous flow near the tube. PGAs, prostaglandin analogs; CAIs, Carbonic anhydrase inhibitors.

Notably, the MTMT group showed a remarkable reduction in ECD (−1,050 cells/mm^2^/year), which was insignificant compared with the AGV group, but was significant compared with the baseline. The ECD changes with MTMT in glaucoma patients who underwent PKP have yet to be reported probably due to the failure to consider reduction in ECD following MTMT. The effect of topical CAIs on ECD remains controversial. Some clinical studies showed no significant reduction in ECD following the application of topical CAIs in patients with glaucoma who did not undergo intraocular surgery although corneal thickness was increased occasionally (25, 26). The other studies show that CAI effect on ECD is primarily related to attenuation of the bicarbonate efflux (3, 4). Eye bank data showed no significant correlation of ECD loss with anti-glaucoma medication in the absence of ocular surgery (8). However, the ECD of the donor graft in recipients after PKP may be affected differently by anti-glaucoma medications. The annual rate of endothelial cell loss is 0.6% in the normal adult human cornea (15). By contrast, the compromised cornea after PKP loses endothelial cells at a rate of 7.8–7.9% per year over 10 years (14, 27, 28). The endothelial cells can be significantly affected by topical CAIs in compromised corneas with guttata (29, 30). Therefore, the long-term MTMT using topical CAIs may affect ECD in compromised corneas as in PKP. In line with this, our study suggested that ECD may be significantly reduced by the long-term MTMT in patients who underwent PKP. Another possibility is that the long-term exposure to higher IOP in MTMT compared with AGV implantation may cause a reduction of ECD. Meanwhile, MTMT did not show any differences in hexagonality and CV. It indicates that the functional attenuation may be less in MTMT when compared with AGV implantation despite the reduction of ECD. Besides, the prevalence of superficial punctate epithelial keratopathy in patients with MTMT was also higher than in patients with AGV. Therefore, we hypothesized how to lose endothelial cells with either MTMT or glaucoma drainage device (Figure 6). With long-term anti-glaucoma medication, endothelial toxicity related to low pH, preservatives of anti-glaucoma eyedrops, ocular inflammation related to an ingredient, such as prostaglandin analogs, or CAIs' effects on corneal endothelial transports may contribute to the reduction of endothelial cell density in corneal grafts (Figure 6, upper chart) (30–34). Whereas, turbulent aqueous flow or mechanical stress near the tube seems to contribute directly in the reduction of endothelial cell density in corneal grafts (Figure 6, lower chart) (35). Long-term graft survival with the shunt surgery was known to be lower than that with anti-glaucoma medication (6), whereas damaged ocular surface may end up with poor compliances, and it can induce less-controlled IOP along with the reduced graft survival in MTMT. Given that the prevalence of SPK was more than double in MTMT, although it was not significant, increased SPK may contribute to further ocular surface inflammation or may affect drug compliance. Therefore, AGV implantation can be still considered as an alternative option for patients who require long-term MTMT in the compromised cornea with PKP.

Besides, current studies investigated the effect of minimally invasive glaucoma surgery (MIGS) on corneal endothelial cells. The CyPass supraciliary microshunt (Alcon Laboratories, Fort Worth, TX) has been withdrawn from the global marketplace because of its adverse effect on ECD (36, 37). It has been reported that endothelial cell loss at 24 months after the XEN gel stent (Allergan, Belfast, Ireland) implantation was 14.5% (38). As for the Ex-Press mini-shunt (Alcon Laboratories), endothelial cell loss at 12 months was 10.0% and at 24 months was 18.0% in long-term studies (39, 40). There are only several case reports describing the effects of MIGS on corneal endothelial cells in post-keratoplasty glaucoma so far, and the results are controversial (41, 42).

The study was limited by the small number of cases, the retrospective study design, and the short-term outcome based on 1-year data analysis. Due to the retrospective nature, the study design was failed to show direct head-to-head comparison and randomization of MTMT or AGV implantation was unavailable due to an ethical issue. Although not statistically significant, the mean age of patients in the MTMT group was almost 5 years older than that of the AGV group, pre-operative IOP was high in the AGV group, and the time between PKP and onset of anti-glaucoma treatment was almost double that of the MTMT group. These differences may have affected the corneal endothelial cells loss. For the survival analysis, limited follow-up and different follow-up duration may have increased the risk of bias in the comparative survival analysis. Finally, re-PKP in both groups, which was insignificant, may also have affected the survival of the corneal grafts. Therefore, further long-term prospective study is pending.

Although this study is preliminary, it is still worthwhile to reveal the possible endothelial toxicity with long-term MTMT in transplanted corneal graft for previous bullous keratopathy. It indicates that an alternative option with an AGV implantation or MIGS may be required in patients with PKP when the long-term MTMT is mandatory. In summary, MTMT seems to affect the endothelial cell density in patients who underwent PKP for bullous keratopathy, and it appears to be similar to the initial effect of AGV implantation. Therefore, careful monitoring of ECD should be considered in corneal transplanted patients with MTMT for glaucoma.

## Data Availability Statement

The data analyzed in this study is subject to the following licenses/restrictions: The datasets contains patient information and is not publicly available. Requests to access these datasets should be directed to Mee Kum Kim, kmk9@snu.ac.kr.

## Ethics Statement

The studies involving human participants were reviewed and approved by Institutional Review Board of Seoul National University College of Medicine. Written informed consent for participation was not required for this study in accordance with the national legislation and the institutional requirements.

## Author Contributions

MK is the principal investigator of the study and made substantial contributions to the design for this study and participated in the interpretation of data and the critical revision of the manuscript for important intellectual content. SW made substantial contributions to the acquisition of data and analysis of data for this study and drafted the article. YK and JJ participated in the interpretation of data and the revision of the manuscript. All authors read and approved the final manuscript.

## Conflict of Interest

The authors declare that the research was conducted in the absence of any commercial or financial relationships that could be construed as a potential conflict of interest.

## Publisher's Note

All claims expressed in this article are solely those of the authors and do not necessarily represent those of their affiliated organizations, or those of the publisher, the editors and the reviewers. Any product that may be evaluated in this article, or claim that may be made by its manufacturer, is not guaranteed or endorsed by the publisher.
